# Targeting the brain 5-HT7 receptor to prevent hypomyelination in a rodent model of perinatal white matter injuries

**DOI:** 10.1007/s00702-022-02556-8

**Published:** 2022-11-06

**Authors:** Cindy Bokobza, Alice Jacquens, David Guenoun, Blandine Bianco, Anne Galland, Maxime Pispisa, Alexandra Cruz, Manuela Zinni, Valérie Faivre, Anne Roumier, Sophie Lebon, Tania Vitalis, Zsolt Csaba, Tifenn Le Charpentier, Leslie Schwendimann, Pierrette Young-Ten, Vincent Degos, Patricia Monteiro, Pascal Dournaud, Pierre Gressens, Juliette Van Steenwinckel

**Affiliations:** 1 Université Paris Cité, Inserm, NeuroDiderot, 75019 Paris, France; 2 Department of Anesthesia and Critical Care, APHP-Sorbonne University, Hôpital La Pitié- Salpêtrière, Paris, France; 3 Department of Pharmacy, APHP, Hôpital Robert Debré, Université de Paris, Paris, France; 4 Life and Health Sciences Research Institute (ICVS), School of Medicine, University of Minho, Braga, Portugal; 5 Sorbonne Université, Inserm, UMR-S 1270, Paris, France

**Keywords:** Preterm birth, Neurodevelopmental disorders, Serotonin, HTR7, Microglia, Astrocyte, Myelination

## Abstract

Approximately 15 million babies are born prematurely every year and many will face lifetime motor and/or cognitive deficits. Children born prematurely are at higher risk of developing perinatal brain lesions, especially white matter injuries (WMI). Evidence in humans and rodents demonstrates that systemic inflammation-induced neuroinflammation, including microglial and astrocyte reactivity, is the prominent processes of WMI associated with preterm birth. Thus, a new challenge in the field of perinatal brain injuries is to develop new neuroprotective strategies to target neuroinflammation to prevent WMI. Serotonin (5-HT) and its receptors play an important role in inflammation, and emerging evidence indicates that 5-HT may regulate brain inflammation by the modulation of microglial reactivity and astrocyte functions. The present study is based on a mouse model of WMI induced by intraperitoneal (i.p.) injections of IL-1β during the first 5 days of life. In this model, certain key lesions of preterm brain injuries can be summarized by (i) systemic inflammation, (ii) pro-inflammatory microglial and astrocyte activation, and (iii) inhibition of oligodendrocyte maturation, leading to hypomyelination. We demonstrate that *Htr7* mRNA (coding for the HTR7/5-HT7 receptor) is significantly overexpressed in the anterior cortex of IL-1β-exposed animals, suggesting it as a potential therapeutic target. LP-211 is a specific high-affinity HTR7 agonist that crosses the blood–brain barrier (BBB). When co-injected with IL-1β, LP-211 treatment prevented glial reactivity, the down-regulation of myelin-associated proteins, and the apparition of anxiety-like phenotypes. Thus, HTR7 may represent an innovative therapeutic target to protect the developing brain from preterm brain injuries.

**Supplementary Information:**

The online version contains supplementary material available at 10.1007/s00702-022-02556-8.

## Background

Preterm birth represents one of ten births worldwide and is the most prominent cause of death among newborns. A large portion of survivors present diffuse white matter injuries (WMI) (Hinojosa-Rodriguez et al. 2017; Mantoo et al. 2021; Young et al. 2018). Maternal infection is found in 30% of spontaneous preterm deliveries (Goldenberg et al. 2008), taking the form of intra-amniotic infections, uterine infections, or systemic infections (reviewed in Stinson and Payne (2019)). Such infections increase local maternal pro-inflammatory cytokine levels in the cervicovaginal fluid and/or systemically in the plasma (Gillespie et al. 2022; Wei et al. 2010; Kim 2020). This elevation in the levels of circulating inflammatory molecules can induce systemic fetal inflammation (Gomez et al. 1998; Basu et al. 2015), leading to neuroinflammation (Pogledic et al. 2014). Preterm birth and perinatal neuroinflammation are recognized risk factors for the development of several neurodevelopmental disorders (NDDs), such as autism spectrum disorder (ASD), attention-deficit/hyperactivity disorder (ADHD), learning disabilities, intellectual disability, and cerebral palsy (Bokobza et al. 2019; Krakowiak et al. 2017; Zerbo et al. 2014; Moster et al. 2008).

WMI are characterized by a blockade in the maturation of oligodendrocytes, leading to delayed myelination (Favrais et al. 2011). Neuroinflammation associated with WMI is due to the excessive reactivity of glial cells: microglia and astrocytes (Pogledic et al. 2014; Verney et al. 2012; Steenwinckel et al. 2019; Supramaniam et al. 2013; Vontell et al. 2013; Shiow et al. 2017; Wisnowski et al. 2014). Microglia are the brain resident macrophages that react in response to any environmental modification or breakdown in homeostasis. In preterm birth, pro-inflammatory microglial reactivity has been shown to be the key inducer of WMI (Steenwinckel et al. 2019; Krishnan et al. 2017). Similarly, astrocyte reactivity is found in post-mortem tissues from patients with WMI, and targeting astrocytes can prevent such injuries (Shiow et al. 2017; Nobuta et al. 2012). Reactive microglia and astrocytes release numerous pro-inflammatory molecules, such as cytokines and reactive oxygen species (ROS), which can affect oligodendrocyte differentiation and myelination (Chhor et al. 2013; Liddelow et al. 2017; Pang et al. 2000; Brown 2007; Cooney et al. 2013). Moreover, oligodendrocytes can also participate in neuroinflammation, contributing to their own differentiation blockade (Boccazzi et al. 2021). Furthermore, pro-inflammatory microglia disengage from their normal developmental functions (Steenwinckel et al. 2019; Krishnan et al. 2017). In particular, there is a reduction in insulin-like growth factor 1 (IGF1) production by microglia exposed to perinatal inflammation. This cytokine normally supports oligodendrocyte differentiation (reviewed in Steenwinckel et al. (2019); Tilborg et al. 2018). Thus, immature oligodendrocytes are subjected to two detrimental events that block their differentiation: exposure to pro-inflammatory molecules and a reduction in IGF-1 levels. Overall, these studies demonstrate strong crosstalk between microglia, astrocytes, and oligodendrocytes in the etiology of perinatal WMI, suggesting that modulating one of these cell types could lead to a better neurological outcome (Steenwinckel et al. 2019; Shiow et al. 2017; Bokobza 2021).

Serotonin (5-hydroxytryptamine [5-HT]), an amine of the indolamine family, is a very common neurotransmitter, neuromodulator, and hormone. Through 14 receptor subtypes, 5-HT participates in multiple physiological functions, such as thermoregulation, sleep, nociception, intestinal peristalsis, platelet function, vascular tone modification, and smooth muscle tone. 5-HT is also known to be an immuno-regulatory molecule of the peripheral immune system (reviewed in Wu et al. (2019)). For example, 5-HT deficiency in mice has been shown to be associated with the modulation of T-cell activation (Li et al. 2011). Within the central nervous system (CNS), serotonin is produced by serotoninergic neurons located in the raphe nuclei of the brainstem. Accumulating evidence demonstrate that 5-HT and its receptors play an important role in NDD etiology. Alteration of the serotoninergic system may affect developmental neuro-immune interactions, inducing neural connectivity defects and the onset of NDDs (Homberg et al. 2013; Hanswijk, et al. 2020; Ames et al. 2021; Abdulamir et al. 2018; Jaiswal et al. 2015). Moreover, several 5-HT receptors (HTRs) are present in both microglia and astrocytes and modulate neuroinflammation in various pathologies (Bechade et al. 2021; Kolodziejczak et al. 2015; Pinna et al. 2021; Muller et al. 2021; Yun et al. 2015; Yue et al. 2019).

**Fig. 1:**
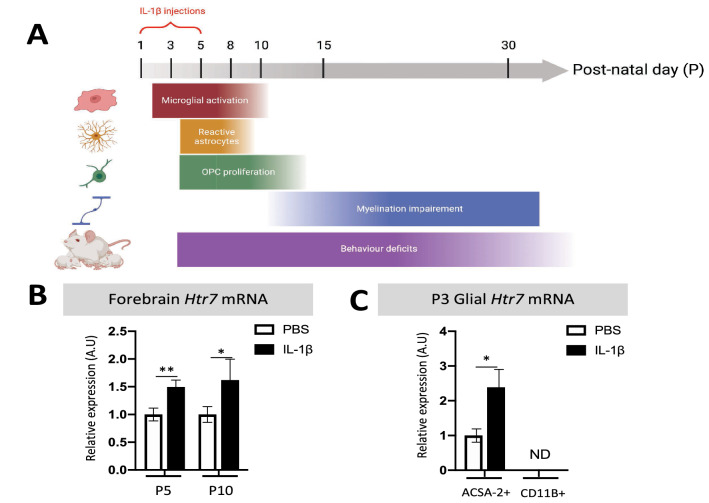
Impact of IL-1β on the expression of *Htr7* mRNA. **A** Recapitulative schematic representation of the perinatal inflammation model induced by IL-1β-exposure and its characteristics. **B** Relative expression of *Htr7* mRNA in the forebrain at P5 and P10 after perinatal administration of PBS or IL-1β. *t* test (*n* = 9–14/group, mean SEM), (*p ≤ 0.05, **p ≤ 0.01, comparing IL-1β vs PBS). **C** Relative expression of *Htr7* mRNA in sorted astrocytes (ACSA-2^+^) and microglia (CD11B^+^) at P3 after perinatal administration of PBS or IL-1β. ND = non-detectable signal. RT-qPCR results are presented as the fold change relative to PBS-exposed animals. T test (*n* = 8/group, mean SEM), (*p ≤ 0.05, comparing IL-1β vs PBS)

5-HT receptor subtype 7 (HTR7) belongs to the G protein-coupled receptor family. During development, HTR7 is strongly expressed in several critical brain regions, such as the pre-frontal cortex, hippocampus, and thalamus (Bokobza et al. 2019; Martin-Cora and Pazos 2004). HTR7 is largely expressed by neurons (Ciranna and Catania 2014), but *Htr7* mRNA has also been detected in microglia and astrocytes (Hirst et al. 1997; Krabbe et al. 2012). Several studies have shown that targeting HTR7 with an agonist or antagonist can improve behavior in NDD animal models (reviewed in Lee, et al. (2021)). In 2008, a brain penetrant agonist of HTR7, N-(4-cyanophenylmethyl) -4-(2-diphenyl)-1-piperazinehexanamide (LP-211), was reported, with a high specific affinity for HTR7 and a relatively low affinity for the 5-HT1A and dopamine D2 receptors (Leopoldo et al. 2008; Hedlund et al. 2010). LP-211 appears to ameliorate neurite outgrowth and behavioral deficits associated with NDD, both in rats and mice (stereotypy, memory, exploration, reviewed in Romano et al. (2014)). LP-211 has since been assessed in several in vivo studies as a promising therapeutic compound to reduce neuroinflammation processes by decreasing mitochondrial ROS production in two Rett syndrome mouse models (Valenti et al. 2017). Therefore, LP-211 could modulate neuroinflammation through the activation of HTR7, either on microglia or astrocytes, and prevent WMI. To test this hypothesis, our group has developed a model of WMI induced by perinatal inflammation using interleukin (IL)-1β. Intra-peritoneal (i.p.) injections of IL-1β during the first 5 days of life can recapitulate certain key events of perinatal WMI: (i) pro-inflammatory microglial and astrocyte activation, (ii) inhibition of oligodendrocyte maturation, leading to hypomyelination, and (iii) behavioral deficits (recapitulated in Fig. 1A) (Favrais et al. 2011; Steenwinckel et al. 2019; Shiow et al. 2017; Bokobza 2021).

The present study aims to evaluate HTR7 activation using LP-211 as a novel therapeutic compound to prevent perinatal brain injuries in our WMI model.

## Methods

### Animals

All animals were handled according to the institutional guidelines of the Institut National de la Santé et de la Recherche Scientifique (Inserm, France). All protocols were approved by the ethical committee of Paris Nord and the Ministry of Research (APAFIS#18422-2019010820435001). A gender bias is present in NDD, since boys are more affected than girls (Bokobza et al. 2019). Therefore, only male pups of the OF1 strain purchased from Charles River Laboratories (L’Abresle, France) were injected i.p. twice daily from postnatal day (P)1 to P5 with recombinant mouse IL-1β (10 μg.kg-1, 130–101-684, Miltenyi Biotec^®^) diluted in 0.12 M 1X PBS (14,200,067, Thermo Scientific) or the same volume of 1X PBS (Steenwinckel et al. 2019). LP-211 (1 mg.kg-1, SML-1561, Sigma-Aldrich^®^) or AS-19 (1 mg.kg-1, 1968/10, Tocris^®^) was co-injected with IL-1β diluted in 0.12 M 1X PBS and 7% DMSO.

### Magnetic-activated cell sorting

At P3, mice were anesthetized with an i.p. injection of pentobarbital (150 mg/kg) and intracardially perfused with 0.9% NaCl. After removal of the olfactive bulbs and cerebellum, tissues were dissociated using the Neural Tissue Dissociation Kit and a gentleMACS Octo Dissociator with the manufacturer's heater (130-092-628, 130-096-427, Miltenyi Biotec^®^). Microglial cells and astrocytes were enriched from the resulting brain homogenate after dissociation using anti-CD11B and anti-ACSA-2 antibodies, respectively, coupled to microbeads according to the manufacturer’s protocol (130-093-634, 130-097-678, Miltenyi Biotec^®^). After elution, 80,000 isolated cells were resuspended in HBSS (14,065,049, Sigma–Aldrich^®^) with calcium and magnesium and distributed in a 96-well plate to measure ROS production (see below). The remaining isolated cells were pelleted and stored at − 80 °C prior to RNA extraction.

### Reactive oxygenate species (ROS) release

ROS release for each sample (20,000 cells/well) was measured in duplicate, either under basal conditions or stimulation (treatment with 10^–7^ M phorbol 12-myristate 13-acetate [PMA, Sigma]). After the addition of luminol (50 µM, Sigma) for 10 min at 37 °C in the dark, luminescence was measured using a Centro LB 960 Microplate Luminometer (Berthold Technologies^®^) and analyzed using MikroWin 2010 software. The signal was recorded at the end of every 3 min cycle over a 20 min period as relative light units. The results were analyzed from the area under the curve of luminescence over the 20 min and are presented relative to control values.

### RNA extraction and quantification of gene expression by real-time qPCR

mRNA from sorted cells and the anterior cerebrum was extracted using NucleoSpin RNA XS Plus and the NucleoSpin RNA set for NucleoZOL (740,990.50, Macherey–Nagel^®^), respectively, according to the manufacturer’s instructions and diluted in 16 or 100 μL RNase-free water, respectively. mRNA was subjected to reverse transcription using iScriptTM cDNA synthesis kit (1,708,890, Bio-Rad^®^). qPCR was performed on selected genes (Table 1) and analyzed using *Rpl13a* mRNA as the reference gene as previously described (Steenwinckel et al. 2019).

**Table 1:** Real-time quantitative PCR (RT-qPCR) primer sequences

Gene	Target protein and abbreviation	Forward	Reverse
*Rpl13a*	Ribosomal protein L13 a	ACA GCC ACT CTG GAG GAG AA	GAG TCC GTT GGT CTT GAG GA
*Htr7*	5-Hydroxytryptamine receptor 7	CATGCACGAAGCCCTGAAAC	TCCCACAGTGGTCACAGTTTT
*Aspg*	Asparaginase	GGCAGGCATCAGAGTGTCAT	TTCGCTGTCTCCCCTTGATG
*Cd44*	Cell Surface Glycoprotein 44	TAGCTGGACACTCAAGTGCG	AGACGGCAAGAATCAGAGCC
*Gfap*	Glial fibrillary acidic protein	AAGCCAAGCACGAAGCTAAC	CTCCTGGTAACTGGCCGACT
*Amigo2*	Adhesion molecule with Ig like domain 2	CCGATAACAGGCTGCTGGAG	AGAATATACCCCGGCGTCCT
*Serping1*	Serpin family G member 1	GCCTCGTCCTTCTCAATGCT	CGCTACTCATCATGGGCACT
*Ugt1a*	UDP Glucuronosyltransferase Family 1 member a complex locus	CTATGTCAACGCCTCTGGGG	GGTCTAGTTCCGGTGTAGCG
*Cd109*	Cluster of differentiation 109	TCCCACTGTGAGAGACTACAAA	ACCTGGGTGTTGTAGCTTCG
*Emp1*	Epithelial membrane protein 1	CTCCCTGTCCTACGGCAATG	GAGCTGGAACACGAAGACCA
*Tm4sf1*	Transmembrane 4 L six family member 1	GGGTTTGGCAGAAGGACCAA	TGCTTGGGCTCATAGCACTT
*Nos2*	Nitric oxide synthase 2	CCC TTC AAT GGT TGG TAC ATG G	ACA TTG ATC TCC GTG ACA GCC
*Ptgs2*	Prostaglandin-endoperoxide synthase 2 (=COX-2)	TCA TTC ACC AGA CAG ATT GCT	AAG CGT TTG CGG TAC TCA TT
*Tnf*	Tumor necrosis factor	GCC TCT TCT CAT TCC TGC TT	AGG GTC TGG GCC ATA GAA CT
*Il1rn*	Interleukin 1 receptor antagonist	TTG TGC CAA GTC TGG AGA TG	TTC TCA GAG CGG ATG AAG GT
*Il4ra*	Interleukin 4 receptor alpha	GGA TAA GCA GAC CCG AAG C	ACT CTG GAG AGA CTT GGT TGG
*Socs3*	Suppressor of cytokines 3	CGT TGA CAG TCT TCC GAC AA	TAT TCT GGG GGC GAG AAG AT
*Mrc1*	Cluster of differentiation 206	CTT CGG GCC TTT GGA ATA AT	TAG AAG AGC CCT TGG GTT GA
*Lgals3*	Lectin galactoside-binding soluble 3	GAT CAC AAT CAT GGG CAC AG	ATT GAA GCG GGG GTT AAA GT
*Igf1*	Insulin-like growth factor 1	TGG ATG CTC TTC AGT TCG TG	GCA ACA CTC ATC CAC AAT GC
*Mbp*	Myelin Basic Protein	CCG GAC CCA AGA TGA AAA C	CTT GGG ATG GAG GTG GTG T
*Mog*	Myelin-oligodendrocyte glycoprotein	AAG AGG CAG CAA TGG AGT TG	GAC CTG CAG GAG GAT
*Plp*	Proteolipid protein	CCA AAT GAC CTT CCA CCT GT	CGA AGT TGT AAG TGG CAG CA

### Brain sections and immunohistochemistry

At P30, brains were processed to paraffin-embedded sections by immediate immersion in 4% formaldehyde for 1 week at room temperature before dehydration and paraffin embedding. The paraffin-embedded samples were then cut into 16 μm sections using a microtome. Immunostaining was performed, as previously described, using a mouse antibody to PLP (28,486, Abcam^®^, 1:500) (Favrais et al. 2011). Images were acquired using a Nikon Eclipse E200 at × 20 microscope. The intensity of the PLP immunostaining in the anterior corpus callosum and somato-sensorial cortex was assessed by densitometric analysis using NIH ImageJ Software (http://imagej.nih.gov/ij/) (Schindelin et al. 2012). The optical density was deduced from the greyscale standardized to the photomicrograph background. One measurement per section (a 4000 mm^2^ area) and four sections were analyzed for each brain.

### Plasma collection and Luminex assay

Blood from animals was collected using a Microvette CB 300 Lithium Heparin collection tube (MVCB-H-300, SAI Infusion^®^). After centrifugation, plasma was collected to perform a Bio-Plex Pro Mouse Cytokine 8-plex Assay (M60000007A, Bio-Rad^®^) according to the manufacturer’s instructions.

### Extraction of proteins and western blotting

Western blot analysis of MBP was performed on protein lysates from the frozen anterior cerebrum at P10 as previously described (Steenwinckel et al. 2019). Briefly, proteins were extracted using RIPA Buffer (R0278, Sigma-Aldrich^®^) containing protease inhibitors (04,693,159,001, Complete Tablets, Roche^®^) in gentleMACS M tubes using a gentleMACS dissociator (Miltenyi Biotec^®^). After centrifugation (10,000 × g, 10 min, 4 °C), the total protein concentration was evaluated using BCA protein assays (BCA1-1KT, Sigma-Aldrich^®^). Samples were then diluted with Laemmli sample buffer (1,610,747, Bio-Rad) containing 2-mercaptoethanol (60-24-2, Sigma–Aldrich^®^) and equal amounts of protein (30 ug) were separated by electrophoresis and electro-transferred onto a 0.2 nitrocellulose membrane. Membranes were blocked for 1 h and incubated overnight with mouse anti-β-actin (Sigma-Aldrich^®^ AC-74, 1:20,000) and rat anti-MBP (Millipore^®^ MAB386 1:500). After rinsing, membranes were incubated for 1 h with an HRP-conjugated goat anti-mouse IgG (1:2,000; Sigma-Aldrich^®^) or HRP-conjugated goat anti-rat IgG (1:10,000; Invitrogen^®^) in blocking solution. Immuno-labeling was revealed using ClarityTM Western ECL substrate (Bio-Rad^®^) and blot images were acquired using Syngene PXi (Syngene^®^) software. The immunoreactivity of four isoforms of MBP was compared to that of actin controls using NIH ImageJ software (http://rsb.info.nih.gov/ij/).

### Slice preparation and compound action potential (CAP) electrophysiological recording

Coronal brain slices (Bregma 0.86–0.02 mm), which included the corpus callosum, were prepared from P30-P35 mice as described previously (Crawford et al. 2009; Baker et al. 2002; Reeves et al. 2005; Monteiro et al. 2018; Ting et al. 2014). Briefly, mice exposed to either PBS or IL-1β $\pm$ LP-211 were deeply anesthetized with avertin i.p. injection (tribromoethanol, 20 mg/mL, 0.5 mg/g body weight) and intracardially perfused with NMDG solution (in mM: NMDG 92, KCl 2.5, NaH_2_PO_4_ 1.2, NaHCO_3_ 30, HEPES 20, Glucose 25, Sodium ascorbate 5, Thiourea 2, Sodium pyruvate 3, MgSO_4_.7H_2_O 10, CaCl_2_.2H_2_O 0.5; pH 7.3–7.4; 300–310 mOsm; saturated with a 95% O_2_/5% CO2 gas mixture). Brains were quickly removed, sliced (0.3 mm) in oxygenated NMDG solution, and placed at 37 °C for recovery. After 10 min, slices were transferred to carbogenated aCSF (in mM: NaCl 119, KCl 2.5, NaH_2_PO_4_ 1.2, NaHCO_3_ 24, Glucose 12.5, MgSO_4_.7H_2_O 2, CaCl_2_.2H_2_O 2; pH 7.3–7.4; 300–310 mOsm; saturated with 95% O2/5% CO2 gas mixture) and allowed to rest at room temperature (22–23 °C) for at least 1 h before recordings. Two to three slices per animal were used for compound action potential (CAP) electrophysiological recordings. Briefly, each slice was individually transferred to the recording chamber, perfused at a rate of 1–2 ml/min with carbogenated aCSF, and visualized using an Olympus BX-51WI microscope with IR-DIC (infrared-differential interference contrast). Stimulation was performed using an Isoflex stimulator (AMPI) connected to a platinum–iridium concentric bipolar electrode (CBAPC75, FHC) that was placed in the corpus callosum (approximately 0.5 mm lateral to the midline). Borosilicate glass microelectrodes (GB150F-8P, Science Products) were pulled on a P-97 horizontal puller (Sutter Instruments), backfilled with 3 M NaCl, and placed in the corpus callosum of the contralateral hemisphere at a distance of approximately 1.0 mm from the stimulating electrode. Standard input–output functions were generated for each slice through consecutive stimulation rounds from 0.0 to 4.0 mA in 0.2 mA increments (triplicate measurements per stimulation intensity). The amplitude of the negative peak 1 (N1) and N2 was determined by the average amplitude from the triplicated measurements per stimulation intensity. Signals were amplified using a MultiClamp 700B and sampled at 10 kHz using a Digidata 1440A acquisition system. Analysis was performed using pCLAMP 10 software (Axon Instruments/Molecular Devices).

### Open-field test

Two-month-old animals, exposed to either PBS or IL-1β $\pm$ LP-211, were placed in a 60 × 40 × 22 cm arena for 20 min. Behaviors were recorded and analyzed using ANY-maze software. Spontaneous locomotion was evaluated by the total traveled distance. Anxiety-like behavior was evaluated by the total duration spent at the center stage of the arena.

### Statistical analysis

No statistical methods were used to predetermine sample sizes, but they were similar to those generally used in the field. Data were tested using an unpaired *t* test or by one-way ANOVA. Data handling and statistical processing were performed using Microsoft Excel and GraphPad Prism 8 Software.

## Results

### Perinatal exposition to IL-1β modulates brain *Htr7* mRNA expression

We first evaluated the therapeutic potential of HTR7 in perinatal brain injuries by evaluating any modulation of its expression in the forebrain. IL-1β-exposed animals showed significant and persistent overexpression of *Htr7* mRNA, both at P5 and P10, relative to that of PBS-exposed animals (fold change of 1.49 $\pm$ 0.17 and 1.71 $\pm$ 0.34, respectively; Fig. 1B). The main mediators of IL-1β exposure within the brain are microglia and astrocytes, which present reactive phenotypes. We hypothesized that HTR7 on these cells might modulate their reactivity and prevent brain damage, including WMI. We therefore evaluated *Htr7* mRNA expression in magnetically cell-sorted microglia (CD11B^+^ cells) and astrocytes (ACSA-2^+^ cells) at P3, for which we previously demonstrated that microglial reactivity is well established due to IL-1β exposure (Steenwinckel et al. 2019). Astrocytic expression of *Htr7* mRNA was detectable and significantly up-regulated at P3 in IL-1β-exposed animals (fold change of 2.34 $\pm$ 0.51). On the contrary, we were unable to detect any expression of *Htr7* mRNA in microglia at P3 (Fig. 1C). These observations are coherent with the data from glial RNAseq databases (Clarke et al. 2018; Bennett et al. 2016). Astrocytic *Htr7* mRNA overexpression suggests that this receptor may play a role in glial reactivity in the context of perinatal exposition to IL-1β.

### Peripheral HTR7 activation does not modulate inflammation in the plasma

**Fig. 2:**
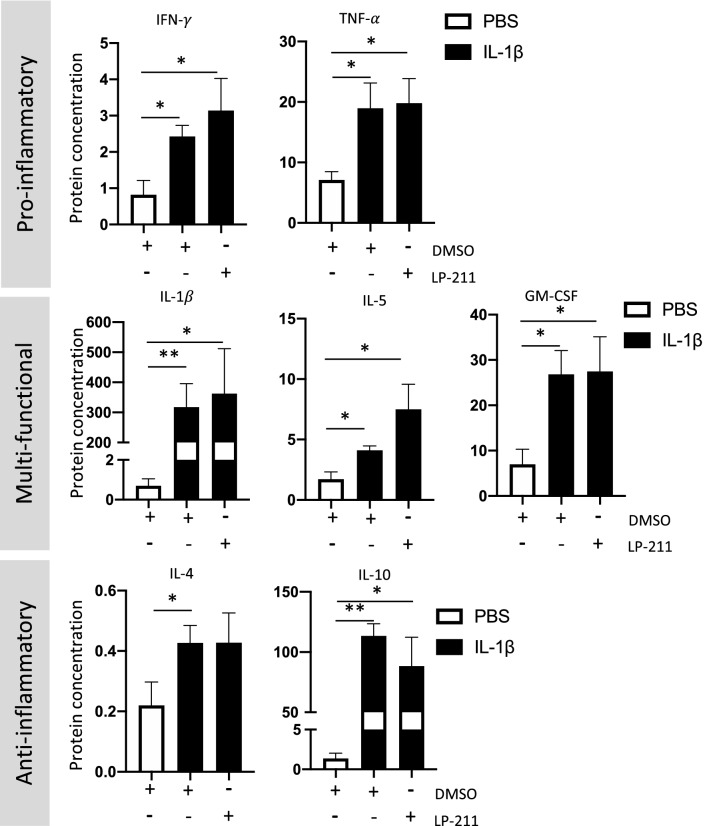
Impact of the agonist LP-211 on plasma cytokine levels at P3 following IL-1β administration. Plasma concentrations of pro-inflammatory (IFN-γ and TNF-α), multi-functional (IL-1β, IL-5, GM-CSF), and anti-inflammatory (IL-4 and IL-10) cytokines, in pg/mL, at P3 after perinatal administration of PBS or IL-1β co-injected with LP-211. One-way analysis of variance (ANOVA) Kruskal–Wallis test corrected by Dunn’s test (*n* = 6/group, mean SEM), (*p ≤ 0.05, **p ≤ 0.01, in comparison to PBS)

In the present model of WMI, IL-1β-administration induces systemic inflammation, as demonstrated by the elevated level of plasma pro-inflammatory cytokines at P5 (Favrais et al. 2011). Systemic inflammation induces neuroinflammation and subsequent WMI (Bokobza et al. 2019; Pinna et al. 2021). As HTR7 is expressed by immune cells, including T cells, neutrophils, and monocytes (Robson et al. 2017), we first evaluated the effect of two HTR7 agonists on systemic inflammation: LP-211, which passes through the blood–brain barrier (BBB), and AS-19, which does not. For both agonists, we evaluated plasma cytokine levels at P3. IL-1β exposure induced overexpression of IFN-γ (fold change of 2.1 $\pm$ 0.62), TNF-α (fold change of 2.7 $\pm$ 0.59), IL-1β (fold change of 228 $\pm$ 56), IL-5 (fold change of 1.6 $\pm$ 0.15), IL-4 (fold change of 1.6 $\pm$ 0.22), IL-10 (fold change of 55 $\pm$ 4.9), and GM-CSF (fold change of 1.9 $\pm$ 0.37) at P3, which were not significantly modulated either by LP-211 (Fig. 2) or AS-19 (Supplementary Material 1).

Overall, these data suggest that HTR7 activation with agonists does not modulate systemic inflammation induced by perinatal neuroinflammation.

### LP-211 prevents glial reactivity induced by IL-1β perinatal exposure

Astrocyte and microglial reactivity can be analyzed using well-described phenotypes (Steenwinckel et al. 2019; Chhor et al. 2013; Liddelow et al. 2017; Bokobza 2021). Reactive astrocytes can be classified (Liddelow’s classification) by transcriptomic profiles into pan-reactive, neurotoxic (A1), and protective (A2) populations (Liddelow et al. 2017). Microglia can also be categorized by transcriptomic phenotypes into pro-inflammatory, anti-inflammatory, and immuno-regulatory activation phenotypes (Steenwinckel et al. 2019; Chhor et al. 2013). IL-1β exposure during the first postnatal week induces microglial and astrocyte reactivity. Microglia present pro-inflammatory and immune-regulatory expression profiles that decrease over time (Steenwinckel et al. 2019). In our model, we previously demonstrated that decreasing glial reactivity ameliorates the myelination deficit and prevents WMI (Steenwinckel et al. 2019; Shiow et al. 2017; Bokobza 2021). In this context, we decided to evaluate the action of LP-211 on glial reactivity by quantifying mRNA marker expression in magnetically cell-sorted astrocytes (ACSA-2^+^) and microglia (CD11B^+^), a hallmark of WMI associated with perinatal inflammation (Steenwinckel et al. 2019; Chhor et al. 2013; Liddelow et al. 2017; Bokobza 2021).

**Fig. 3:**
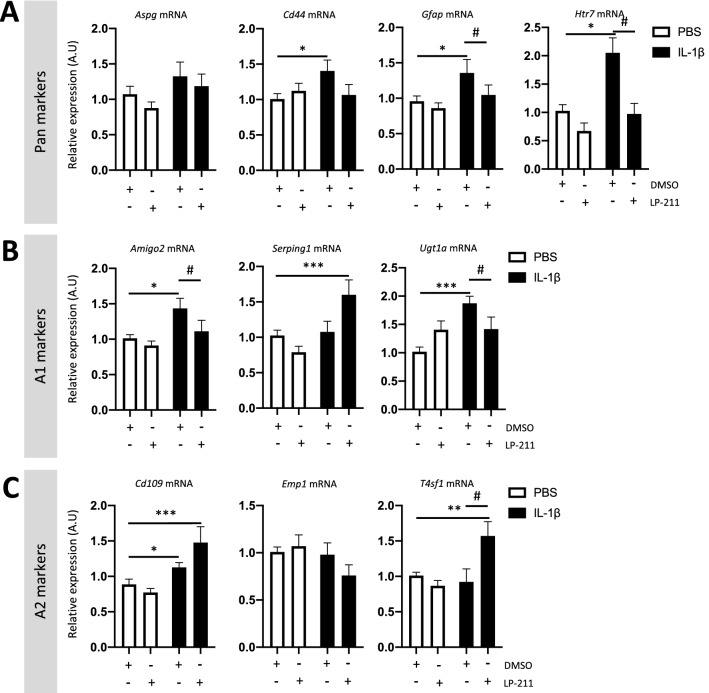
Impact of the agonist LP-211 on astrocyte reactivity at P3 following IL-1β administration. Relative expression of mRNA encoding pan-reactive (**A**), A1- (**B**), and A2-reactive markers (**C**) by astrocytes from P3 animals after perinatal administration of PBS or IL-1β co-injected with LP-211. Cells were obtained after ACSA-2^+^-cell magnetic sorting. RT-qPCR results are presented as the fold change relative to PBS-DMSO-exposed animals. One-way analysis of variance (ANOVA) Kruskal–Wallis test corrected by Dunn’s test (*n* = 9–12/group, mean SEM), (*p ≤ 0.05, **p ≤ 0.01, ***p ≤ 0.001, comparing IL-1β vs PBS, #*p* ≤ 0.05, comparing LP-211 vs DMSO)

As previously described, IL-1β-exposed animals showed astrocytic overexpression of *Htr7* mRNA expression (fold change of 2.053 $\pm$ 0.26) that is significantly counteracted by LP-211 co-treatment (fold change of 0.972 $\pm$ 0.19, Fig. 3A). IL-1β-exposed animals showed overexpression of pan-reactive markers by astrocytes: *Cell Surface Glycoprotein 44 (Cd44* mRNA, fold change of 1.40 $\pm$ 0.15) and *Glial fibrillary acidic protein* (*Gfap* mRNA*,* fold change of 1.35 $\pm$ 0.18); and cytotoxic A1-markers: *Adhesion Molecule with Ig Like Domain 2* (*Amigo2* mRNA, fold change of 1.43 $\pm$ 0.14) and *UDP glucuronosyltransferase family 1 member A complex locus (Ugt1a* mRNA, fold change of 1.871 $\pm$ 0.13). Neither *Asparaginase* (*Aspg*) nor *Serpin family G member 1* (*Serping 1*) mRNA were modulated by IL-1β alone. The pro-regenerative A2-marker *Cluster of differentiation 109* (*Cd109)* mRNA was also modulated by IL-1β (fold change of 1.12 $\pm$ 0.06). LP-211 co-treatment with IL-1β reduced the overexpression of these neurotoxic A1-astrocyte markers and induced the expression of pro-regenerative A2-markers, as demonstrated by the significant overexpression of *Transmembrane 4 L six family member 1* (*T4sf1* mRNA, fold change of 1.57 $\pm$ 0.2, Fig. 3). Interestingly, LP-211 associated with IL-1β induced significant overexpression of *Serping1* mRNA, classically linked to neurotoxicity in the adult brain but not in neonates (Shiow et al. 2017). Liddelow’s classification is based on in vitro and neurodegenerative in vivo models*.* Our results raise questions about the use of Liddelow’s classification in pediatric models (see discussion below).

**Fig. 4:**
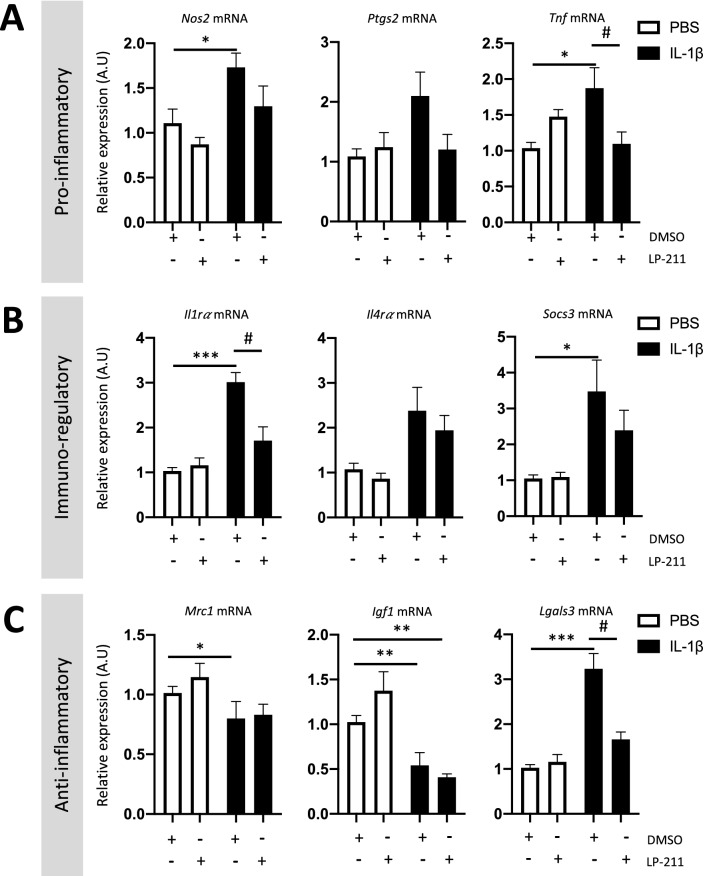
Impact of the agonist LP-211 on microglial reactivity at P3 following IL-1β administration. Relative expression of mRNA encoding pro-inflammatory (**A**), immune-regulatory (**B**), and anti-inflammatory markers (**C**) by microglia from P3 animals after perinatal administration of PBS or IL-1β co-injected with LP-211. Cells were obtained after CD11B^+^-cell magnetic sorting. RT-qPCR results are presented as the fold change relative to PBS-DMSO-exposed animals. One-way analysis of variance (ANOVA) Kruskal–Wallis test corrected by Dunn’s test (*n* = 9–12/group, mean SEM), (*p ≤ 0.05, **p ≤ 0.01, ***p ≤ 0.001, comparing IL-1β vs PBS, ^#^*p* ≤ 0.05, comparing LP-211 vs DMSO)

IL-1β exposure also induced microglial reactivity. Indeed, at P3, CD11B^+^ cells from IL-1β-exposed animals overexpressed pro-inflammatory markers: *Nitric oxide synthase 2* (*Nos2* mRNA, fold change of 1.73 $\pm$ 0.16) and *Tumor necrosis factor* (*Tnf* mRNA, fold change of 1.86 $\pm$ 0.29); and immuno-regulatory markers: *Interleukin 1 receptor antagonist* (*Il-1ra* mRNA, fold change of 3.01 $\pm$ 0.21) and *Suppressor of cytokine signaling 3* (*Socs3* mRNA, fold change of 3.47 $\pm$ 0.88), as previously described (Steenwinckel et al. 2019; Bokobza 2021). Neither *Prostaglandin-endoperoxide synthase 2* (*Ptgs2*) or *interleukin 4 receptor antagonist* (*Il-4ra*) mRNA were modulated by IL-1β. IL-1β also modulated the expression of anti-inflammatory markers in microglia by inducing the overexpression of *galectin-3* (*Lgals3* mRNA, fold change of 3.24 $\pm$ 0.34) and reducing the expression of *Mannose receptor C-type 1* (*Mrc1)* and *Igf1* mRNA (fold changes of 0.80 $\pm$ 0.14 and 0.54 $\pm$ 0.14, respectively), as previously described (Steenwinckel et al. 2019; Bokobza 2021). Treatment with LP-211 significantly decreased the expression of *Tnf, Il-1ra,* and *Lgals3* mRNA relative to IL-1β-exposed animals (Fig. 4).

**Fig. 5:**
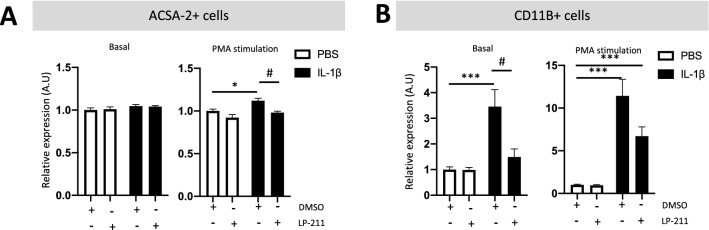
Impact of the agonist LP-211 on glial ROS production at P3 following IL-1β administration. Relative expression of reactive oxygen species (ROS) production by astrocytes (ACSA-2^+^) and microglia (CD11B^+^) from P3 animals after perinatal administration of PBS or IL-1β co-injected with LP-211. Results of the basal and PMA-stimulated states are presented as the fold change relative to PBS-DMSO-exposed animals. One-way analysis of variance (ANOVA) Kruskal–Wallis test corrected by Dunn’s test (*n* = 9–12/group, mean SEM) (*p ≤ 0.05, ***p ≤ 0.001, comparing IL-1β vs PBS, ^#^*p* ≤ 0.05, comparing LP-211 vs DMSO)

ROS are small molecules derived from oxygen that are overproduced within the CNS during neuroinflammation. ROS are harmful to the brain parenchyma due to oxidative stress. Enzymes responsible for the production of ROS include nicotinamide adenine dinucleotide phosphate (NADPH) oxidases (NOX), which are present in neuronal, microglial, and astrocytic membranes (reviewed in Nayernia et al. (2014)). Pharmacologically, it is possible to enhance basal ROS production by stimulating protein kinase C (PKC) with PMA (Bhat et al. 2019). Thus, evaluating ROS production by astrocytes and microglia, both at the basal and PMA-stimulated states, in our WMI model is a relevant functional test to evaluate glial reactivity. At P3, ACSA-2^+^ cells from IL-1β-exposed animals did not produce ROS. However, stimulation with PMA resulted in a significant increase in ROS production in astrocytes from IL-1β-exposed animals (fold change of 1.12 ± 0.03) relative to PBS-exposed animals. LP-211 co-treatment prevented astrocytic ROS overproduction following PMA stimulation, with ROS levels similar to those of PBS-exposed animals (Fig. 5A). CD11B^+^ cells from IL-1β-exposed animals showed ROS overproduction at both the basal and PMA-stimulated states (fold change of 3.45 ± 0.66 and 11.42 ± 1.95, respectively) that was significantly counteracted by LP-211 co-treatment (Fig. 5B).

Collectively, these data show that the HTR7 agonist LP-211 reduces neuroinflammation induced by IL-1β through the modulation of astrocyte and microglial reactivity.

### LP-211 prevents hypomyelination in IL-1β-exposed animals

**Fig. 6:**
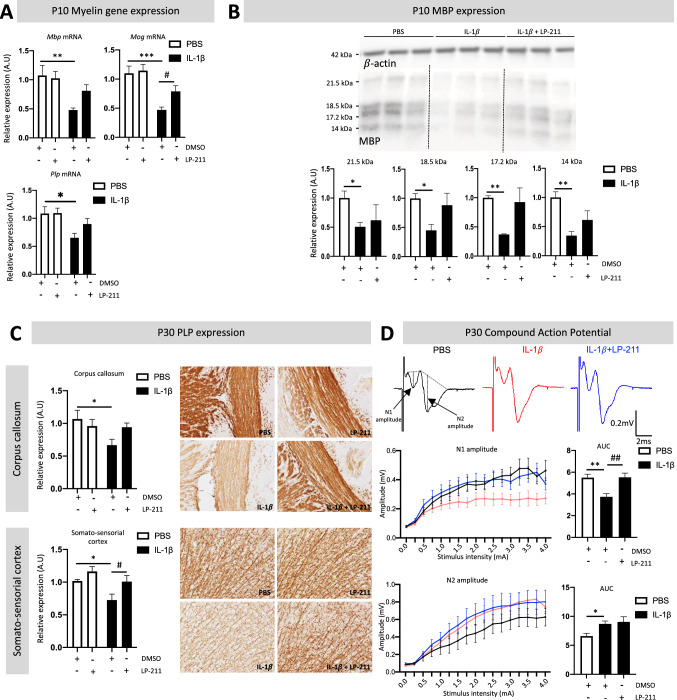
Impact of the agonist LP-211 on myelinization following IL-1β administration. **A** Relative expression of myelin protein mRNA in the forebrain of P10 animals after perinatal administration of PBS or IL-1β co-injected with LP-211. **B** Representative western blots and relative expression of myelin basic protein (MBP) isoforms in the forebrain at P10 after perinatal administration of PBS or IL-1β co-injected with LP-211. One-way analysis of variance (ANOVA) Kruskal–Wallis test corrected by Dunn’s test (*n* = 5–7/group, mean SEM) (*p ≤ 0.05, **p ≤ 0.01, in comparison to PBS). **C** Representative immunohistochemistry images and relative expression of PLP in the corpus callosum and somato-sensorial cortex from P30 animals after perinatal administration of PBS or IL-1β co-injected with LP-211. Results are presented as the fold change relative to PBS-DMSO-exposed animals. One-way analysis of variance (ANOVA) Kruskal–Wallis test corrected by Dunn’s test (*n* = 6/group, mean SEM for RT-qPCR, n = 5–7/group, mean SEM for histology) (*p ≤ 0.05, ***p ≤ 0.001, comparing IL-1β vs PBS, #*p* ≤ 0.05, comparing LP-211 vs DMSO). **D** Representative traces of CAP and measure of N1 and N2 amplitude under increasing stimulation intensity (0–4 mA) in the corpus callosum from P30-35 animals after perinatal administration of PBS or IL-1β co-injected with LP-211. Results are presented as the total area under the curve (AUC). Brown-Forsythe ANOVA test corrected by Dunnett’s T3 test (*n* = 6–8/group, mean SEM), (*p ≤ 0.05, **p ≤ 0.01, comparing IL-1β vs PBS, ^##^*p* ≤ 0.01, comparing LP-211 vs DMSO)

A key mechanism in perinatal WMI is the blockade of oligodendrocyte maturation, leading to delayed myelination (Fig. 1A) (Bokobza et al. 2019; Tilborg et al. 2016). We first characterized the therapeutic effect of LP-211 on myelination by evaluating myelin protein gene expression in the forebrain by qRT-PCR (Fig. 6A). IL-1β induced a significant decrease in the expression of *Mbp, Myelin-oligodendrocyte glycoprotein (Mog)* and *Proteolipid protein 1(Plp)* mRNA at P10 in the anterior cerebrum (fold changes of 0.48 ± 0.04, 0.47 ± 0.05, and 0.65 ± 0.08, respectively). LP-211 treatment prevented the decrease in *Mbp*, *Mog* and *Plp* mRNA expression (Fig. 6A).

MBP is among the most abundant myelin proteins in the CNS (Boggs 2006). We previously demonstrated that evaluation of the four isoforms of MBP in the forebrain by western blotting is a simple and robust method to evaluate the delay in myelination at P10-15 in our model, which is consistent with the reduced myelination observed later (Steenwinckel et al. 2019; Bokobza 2021). We evaluated the effect of LP-211 treatment on delayed myelination induced by IL-1β by quantifying MBP expression in the forebrain at P10 (Fig. 6B). In accordance with previous data, IL-1β exposure induced significant under-expression of MBP isoforms at P10. We observed that LP-211 treatment allowed MBP expression that is not significantly different from the IL-1β-exposed animal but similar to the one in PBS-treated animals. Modulating HTR7 activity only in the periphery using AS-19 was not able to rescue MBP deficits associated with IL-1β exposure (Supplementary Material 2).

PLP is the most abundant transmembrane myelin protein and oversees the correct compaction of myelin sheets (Clarke et al. 2018). Moreover, our group showed that IL-1β exposure induces a reduction in PLP expression at P30 in the cingulum, corpus callosum, and external capsule (Favrais et al. 2011). Thus, evaluating the effect of LP-211 treatment on PLP expression could confirm the protective effect of the HTR7 agonist against WMI. PLP immunochemistry in the corpus callosum and somatosensory cortex at P30 confirmed that animals exposed to IL-1β showed a significant reduction in PLP levels (fold change of 0.67 ± 0.09, and 0.73 ± 0.09, respectively). LP-211 treatment restored PLP expression in both regions similar to that of PBS-exposed animals (Fig. 6C).

One method to evaluate myelin functionality (e.g., acceleration of electric signal propagation) is to record compound action potentials (CAP) (Mu et al. 2019; Li et al. 2016). This method consists in performing extracellular electrophysiological recordings in the corpus callosum to evaluate inter-hemispheric electric conduction. Given that IL-1β exposure induced quantitative myelin deficits, one question that arises pertains to the functionality of myelin following perinatal inflammation. CAP recordings in the corpus callosum of P30-35 PBS-exposed animals highlighted the presence of two negative peaks (N), following the stimulus artifact: the first peak (N1) is correlated to the electric transduction from myelinated axons and the second one (N2) is correlated to the electric transduction from unmyelinated axons. Similar raw traces were obtained in IL-1β-exposed animals co-treated or not with LP-211 (Fig. 6D). However, quantification of negative peak’s amplitudes revealed that the area under the curve (AUC) in PBS-exposed animals for N1 amplitude traces was at 5.486 ± 0.33, whereas IL-1β exposure induced a significant decrease of the N1 amplitude AUC (3.728 ± 0.3) that was significantly counteracted by LP-211 co-treatment (5.517 ± 0.401). In PBS-exposed animals, the N2 amplitude AUC was at 6.598 ± 0.482. We observed that IL-1β exposure induced a significant increase in the N2 amplitude AUC (8.683 ± 0.518). N2 amplitude AUC was, in LP-211-treated animals, not significantly different from IL-1β-exposed animals. Thus, LP-211 corrected N1-associated deficits but not N2-associated deficits (Fig. 6D).

### LP-211 prevents anxiety in IL-1β-exposed animals

**Fig. 7:**
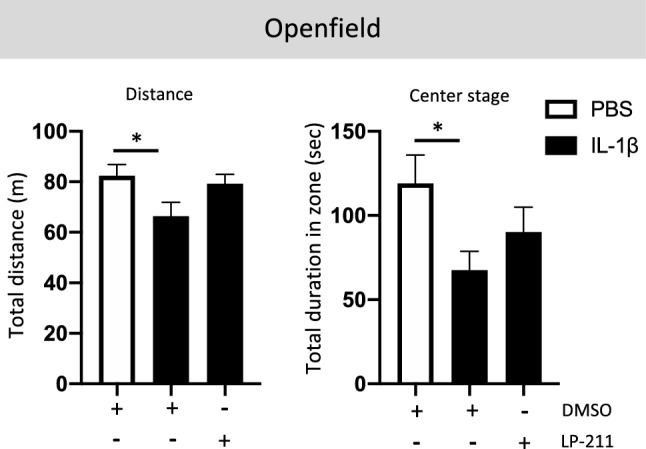
Impact of the agonist LP-211 on anxiety-like behavior following IL-1β administration. Two-month-old animals were submitted to a 20-min open-field test. Travel distance and time spent in the center stage of the arena were measured. One-way analysis of variance (ANOVA) Kruskal–Wallis test corrected by Dunn’s test (*n* = 15/group, mean SEM), (*p ≤ 0.05, comparing IL-1β vs PBS)

Analysis of the behavior of IL-1β-exposed animals at P39 in the elevated plus-maze and open field previously showed an increase in anxiety-like behavior, indicated by significantly less time spent exploring the center zone (Veerasammy et al. 2020). LP-211 has been reported to be a treatment that alleviates behavioral deficits associated with NDDs in a rat model, including anxiety (Khodaverdi et al. 2021). We evaluated the effect of LP-211 on anxiety-related behavior induced by perinatal inflammation by evaluating the spontaneous locomotor activity in 2-month-old animals for 20 min in an open-field test. Perinatal exposition to IL-1β induced a significant decrease in spontaneous locomotor activity evaluated by the total traveled distance over 20 min. In accordance with previous data (Veerasammy et al. 2020), IL-1β-exposed animals spent significantly less time in the center stage of the arena, suggesting anxiety-like behavior. LP-211 co-treatment significantly prevented both locomotor deficits and anxiety-like behavior (Fig. 7).

## Discussion

Preterm birth is a major societal challenge due to the resulting clinical, economic, and, above all, emotional consequences. Among the brain lesions of former preterm infants are those associated with so-called encephalopathy of prematurity (EoP). WMI is the most frequent and still poorly understood lesion in EoP due to the multiplicity of risk factors. Unfortunately, it is still a frequent event associated with prematurity, with a potential lifelong impact. There is currently no treatment to reduce either its incidence or long-term consequences. Here, we evaluated the therapeutic potential of targeting the HTR7 to prevent WMI in a perinatal systemic inflammatory model induced by IL-1β exposure. We first show that *Htr7* mRNA expression is modulated by IL-1β exposure in the forebrain of mice. Glial expression of *Htr7* mRNA was only found and modulated in astrocytes but not in microglia in IL-1β-exposed animals. As astrocyte and microglia reactivity are known to trigger WMI ontogeny, modulating the HTR7 might prevent such deficits. We therefore evaluated the effect of an HTR7 agonist, LP-211, co-injected with IL-1β between P1 and P5. LP-21 was unable to prevent the elevation of plasma cytokine and chemokine levels in IL-1β-exposed animals. LP-211 treatment decreased both astrocyte and microglial reactivity. Furthermore, LP-211 protected the brain against the myelin-associated proteins deficit induced by perinatal inflammation at a later stage. By allowing correct brain myelination, we were able to partially restore a normal electric signal transduction through the corpus callosum. Finally, LP-211 treatment was able to prevent hypolocomotion and anxiety-related behavior induced by perinatal inflammation in young adult mice (P60).

The first finding to emerge from this study was that, in the context of perinatal i.p. administration of the cytokine IL-1β, activation of the HTR7 by two different agonists did not modulate systemic inflammation. This is surprising, as the HTR7 can be found in monocytes (Soga et al. 2007), neutrophils (Robson et al. 2017), and lymphocytes (Leon-Ponte et al. 2007; Urbina et al. 2014). The modulation of HTR7 activity in vitro with agonists and antagonists has highlighted the favorable (de las Casas-Engel et al. 2013; Dominguez-Soto et al. 2017) or detrimental (Soga et al. 2007; Leon-Ponte et al. 2007; Idzko et al. 2004) role of the HTR7 in the peripheral immune system, depending on the study. This is the first study to evaluate the modulation of the peripheral immune system by HTR7 activation in a developmental context. In such a paradigm, the HTR7 did not modulate the release of several cytokines into the blood circulation; this may be partially due to the immaturity of the immune system (Al Nabhani and Eberl 2020).

Astrocyte reactivity is a major component of neuroinflammation. It is described in the literature as a continuum of activation profiles that vary depending on age, stimuli, and/or injury (Escartin et al. 2019). Astrocyte reactivity can be evaluated either by morphological or molecular changes. Here, we evaluated astrocyte reactivity by quantifying mRNA markers that were described in adults by Liddlelow et al. (Liddelow et al. 2017) to be pan-reactive (*i.e.*, constitutively expressed for all stimulation), A1-reactive (*i.e.,* expressed when astrocytes are stimulated with LPS and neurotoxic when applied to neurons), and A2-reactive (*i.e.,* expressed when astrocytes are stimulated with neurotrophic factors and thus considered to be protective). We previously demonstrated that IL-1β-exposure induced overexpression of A2-reactive markers at P5 by ACSA-1^+^ astrocytes (also called GLAST^+^ astrocytes). Here, we observed that ACSA-2^+^ astrocytes (also called ATP1B2^+^ astrocytes) overexpressed pan- and A1-reactive markers at P3 and that LP-211 treatment not only limited the A1 phenotype but also promoted the expression of pro-regenerative A2-reactive markers. Reactive astrocyte classification has been described for adult animals and/or in neurodegenerative contexts (Liddelow et al. 2017; Ito et al. 2019; Reichenbach, et al. 2019; Giovannoni and Quintana 2020). Little is known about the classification of astrocyte reactivity in the immature brain or the different subpopulations of astrocytes (ACSA-1 *vs* ACSA-2 astrocytes) that co-exist during the perinatal period (see discussion of Shiow et al. (2017)). A better characterization of astrocyte reactivity during development and for different subpopulations is therefore necessary for a better comprehension of such mechanisms.

Based on the Human Protein Atlas single-cell database (Karlsson, et al. 2021; Sjostedt, et al. 2020), *HTR7* mRNA is expressed by astrocytes and oligodendrocytes, but not microglia. In our study, we found that at P3, the HTR7 is expressed by astrocytes but not microglia; it is possible that the effect of LP-211 treatment observed on microglia could be associated, at least partially, with its effect on astrocytes. We observed a diminution of the reactivity of both types of glial cells; such inactivation of astrocytes was sufficient to inactivate the microglia at P3 and allowed correct brain myelination. In previously published transcriptomic data of P5 and P10 O4^+^ sorted cells, there was no detectable *Htr7* mRNA (Steenwinckel et al. 2019). The present study thus underscores the interactions between astrocytes, microglia, and oligodendrocytes in the onset of WMI. Indeed, we previously demonstrated that reactive microglia (Steenwinckel et al. 2019) and astrocytes (Shiow et al. 2017) express COX-2, which inhibits oligodendrocyte maturation by activation of the EP1-receptor, leading to a reduction in MBP synthesis (Steenwinckel et al. 2019; Shiow et al. 2017). In vivo treatment using COX-2 specific inhibitors is sufficient to reestablish correct oligodendrocyte maturation, leading to functional brain myelination associated with an improvement in the behavioral deficits induced by IL-1β-exposure (Shiow et al. 2017). Similarly, we demonstrated, here, that LP-211 treatment reduces astrocyte and microglial reactivity induced by IL-1β-exposure, preventing the deficit in myelination, and reducing anxiety-like behavior. To evaluate the functional alteration of myelin under IL-1β-exposure, we performed CAP recordings that confirm, as known in the literature (Crawford et al. 2009; Baker et al. 2002; Reeves et al. 2005; Monteiro et al. 2018; Ting et al. 2014), that myelinated and unmyelinated fibers display distinct conduction velocities of their electrical response. As such, two negative peaks (N) were readily detected in our electrophysiological recordings: the first peak (N1) is correlated to the electric transduction from myelinated axons (increased conduction speed) and the second peak (N2) is correlated to the electric transduction from unmyelinated axons (lower conduction speed). Results revealed that perinatal inflammation reduced the magnitude of the N1 component, suggesting a reduced number of myelinated axons upon IL-1β-exposure. Moreover, co-treatment with LP-211 reestablished the normal amplitude of N1, indicating that it is possible to rescue IL-1β-induced hypomyelination (or avoid the emergence of hypomyelination) by blocking astrocyte and microglial reactivity with LP-211. Perinatal inflammation also increased the magnitude of the N2 component, suggesting an increased number of unmyelinated axons upon IL-1β-exposure. However, co-treatment with LP-211 did not rescue this N2 phenotype. But why LP-211 has such differential effects on myelinated vs unmyelinated axons? One possibility could be the types of axonal fibers being rescued (or spared). The CAP signal is the sum of all single-fiber action potentials that contribute to the signal. The conduction velocity of this signal is influenced not only by the presence or absence of myelin but also by the diameter of the fibers which produce the response. Responses from largest diameter fibers appear first in the signal than smaller diameter fibers. As such, upon LP-211 treatment, larger axons could be preferentially spared or re-myelinated leading to increased amplitude of N1 without noticeable differences on N2 amplitude.

Finally, although we did not study the role of neurons, the HTR7 is expressed by neurons and its role should therefore not be neglected. One can hypothesize that activated 5HT7 receptors could promote direct or indirect molecular pathways that may counteract IL1B-induced central inflammation. In 2014, Ciranna and Catania reviewed the involvement of the HTR7 in the modulation of neuronal and synaptic activity; mostly, they highlighted the association of HTR7 modulation with NDD deficits and autism-like phenotypes (Ciranna and Catania 2014). For example, in a genetic mouse model of Fragile X syndrome, HTR7 activation is able to reverse long-term synaptic depression (Costa et al. 2015; Costa et al. 2012). The HTR7 knock-out mouse presents behavior deficits, such as obsessive–compulsive disorder or social impairment, and the authors suggested that pharmacologically targeting HTR7 could be a useful therapeutic target for such disorders (Hedlund and Sutcliffe 2007).

## Conclusion

We highlight here, for the first time, the concept of the HTR7 regulating brain inflammation in a context of WMI induced by perinatal exposure that models NDDs associated with prematurity, including memory deficits, anxiety-like-behavior, and deficits in social interactions (Favrais et al. 2011; Steenwinckel et al. 2019; Shiow et al. 2017; Bokobza 2021; Veerasammy et al. 2020). Collectively, these data open new perspectives for therapy to protect the developing brain from the adverse effects of early immune activation.

## Supplementary Information

Below is the link to the electronic supplementary material.

Supplementary file1 Impact of the agonist AS-19 on plasma cytokine levels at P3 following IL-1β administration. Plasma concentrations of pro-inflammatory (IFN-γ and TNF-α), multi-functional (IL-1β, IL-5, GM-CSF), and anti-inflammatory (IL-4 and IL-10) cytokines, in pg/mL, at P3 after perinatal administration of PBS or IL-1β co-injected with AS-19. One-way analysis of variance (ANOVA) Kruskal–Wallis test corrected by Dunn’s test (n = 6/group, mean SEM), (*p ≤ 0.05, **p ≤ 0.01, in comparison to PBS). (PDF 133 KB)

Supplementary file2 Impact of the agonist AS-19 on myelinization following IL-1β administration.Representative western blots and relative expression of myelin basic protein (MBP) isoforms in the forebrain at P10 after perinatal administration of PBS or IL-1β co-injected with AS-19. One-way analysis of variance (ANOVA) Kruskal–Wallis test corrected by Dunn’s test (n = 6–7/group, mean SEM), (*p ≤ 0.05, **p ≤ 0.01, in comparison to PBS). (PDF 248 KB)

## Data Availability

Not applicable.

## Abbreviations

5-HT: Serotonin
ADHD: Attention-deficit/hyperactivity disorder
Amigo2: Adhesion molecule with Ig like domain 2
ASD: Autism spectrum disorder
Aspg: Asparaginase
BBB: Blood–brain barrier
Cd109: Cluster of differentiation 109
Cd44: Cell surface glycoprotein 44
CNS: Central nervous system
Emp1: Epithelial membrane protein 1
EoP: Encephalopathy of prematurity
Gfap: Glial fibrillary acidic protein
HTR: 5-HT receptors
HTR7: 5-HT receptor subtype 7
IGF-1: Insulin-like growth factor 1
IL: Interleukin
Il1rn: Interleukin 1 receptor antagonist
Il4ra: Interleukin 4 receptor alpha
Lgals3: Lectin galactoside-binding soluble 3
LP-211: N-(4-cyanophenylmethyl) -4-(2-diphenyl)-1-piperazinehexanamide
MBP: Myelin basic protein
Mog: Myelin-oligodendrocyte glycoprotein
Mrc1: Cluster of differentiation 206
NADPH: Nicotinamide adenine dinucleotide phosphate
NDD: Neurodevelopmental disorders
Nos2: Nitric oxide synthase 2
NOX: NADPH oxidases
P: Post-natal day
PKC: Protein kinase C
Plp: Proteolipid protein
PMA: Phorbol 12-myristate 13-acetate
Ptgs2/COX-2: Prostaglandin-endoperoxide synthase 2 (= Cox2)
ROS: Reactive oxygen species
Rpl13a: Ribosomal protein L13 a
Serping1: Serpin family G member 1
Socs3: Suppressor of cytokines 3
Tm4sf1: Transmembrane 4 L Six Family Member 1
Tnf: Tumor necrosis factor
Ugt1a: UDP Glucuronosyltransferase Family 1 Member A Complex Locus
WMI: White matter injuries
