# Supplementary figures and images for: Targeting the brain 5-HT7 receptor to prevent hypomyelination in a rodent model of perinatal white matter injuries
Source: J Neural Transm (Vienna). 2022 Nov 6;130(3):281–97. doi: 10.1007/s00702-022-02556-8 (PMC10033587; doi:10.1007/s00702-022-02556-8)

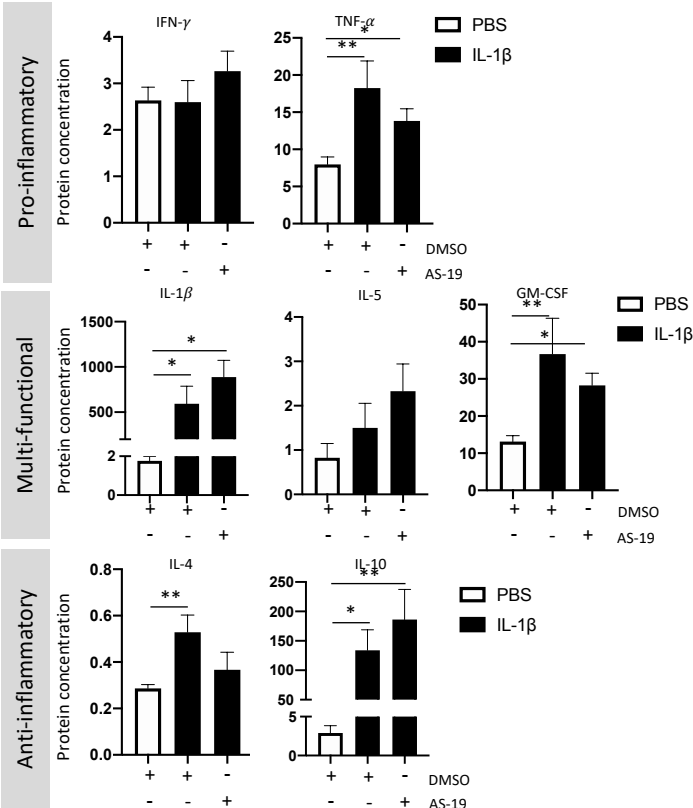

Supplement: Supplementary file 1 — Supplementary file1 Impact of the agonist AS-19 on plasma cytokine levels at P3 following IL-1β administration. Plasma concentrations of pro-inflammatory (IFN-γ and TNF-α), multi-functional (IL-1β, IL-5, GM-CSF), and anti-inflammatory (IL-4 and IL-10) cytokines, in pg/mL, at P3 after perinatal administration of PBS or IL-1β co-injected with AS-19. One-way analysis of variance (ANOVA) Kruskal–Wallis test corrected by Dunn’s test (n = 6/group, mean SEM), (*p ≤ 0.05, **p ≤ 0.01, in comparison to PBS). (PDF 133 KB) [file 702_2022_2556_MOESM1_ESM.pdf]

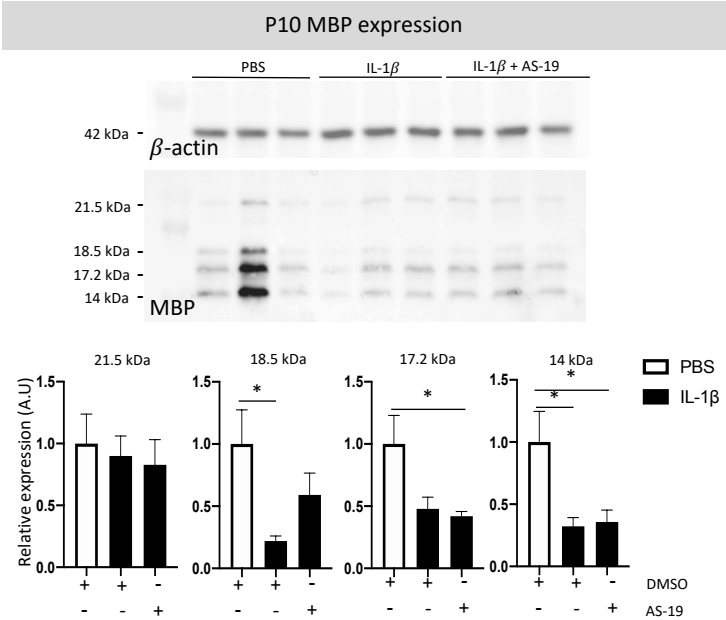

Supplement: Supplementary file 2 — Supplementary file2 Impact of the agonist AS-19 on myelinization following IL-1β administration.Representative western blots and relative expression of myelin basic protein (MBP) isoforms in the forebrain at P10 after perinatal administration of PBS or IL-1β co-injected with AS-19. One-way analysis of variance (ANOVA) Kruskal–Wallis test corrected by Dunn’s test (n = 6–7/group, mean SEM), (*p ≤ 0.05, **p ≤ 0.01, in comparison to PBS). (PDF 248 KB) [file 702_2022_2556_MOESM2_ESM.pdf]
